# Spatio-Temporal Segmentation of Eye Movements During Complex Decision Making Using Switching Hidden Markov Models

**DOI:** 10.3390/jemr19050108

**Published:** 2026-09-22

**Authors:** Anne Guérin-Dugué, Mike Salomone, Aurélie Campagne

**Affiliations:** 1 University Grenoble Alpes, CNRS, Grenoble INP^+^, GIPSA-Lab, ^(+)^Institute of Engineering University Grenoble Alpes, 38000 Grenoble, France; mike.salomone@univ-grenoble-alpes.fr; 2 University Grenoble Alpes, University Savoie Mont Blanc, CNRS, LPNC, 38000 Grenoble, France; aurelie.campagne@univ-grenoble-alpes.fr

**Keywords:** Switching Hidden Markov Model, eye movement, decision making

## Abstract

Understanding how visual exploration evolves over time during complex decision making remains challenging, as the dynamics of underlying cognitive processes are difficult to observe. Hidden Markov models are widely used to model eye movement sequences, but their assumption of stationary transition probabilities limits their ability to identify successive phases. This study investigated whether Switching Hidden Markov Models (SHMMs), trained only on fixation positions, could segment visual exploration during an obstacle-avoidance task requiring a left/right decision. Individual SHMMs with two or three high-level (HI) states were trained without using reaction times. HI states were characterized through transition-matrix variability, saliency maps, cumulative dwell times in regions of interest, and their association with reaction times. Both models produced temporal segmentations consistent with decision making. Early HI states showed greater interindividual variability and exploration focused on obstacles, whereas later states were more homogeneous and reflected decision confirmation followed by reorientation toward the center of the screen. The transition to the final HI state of the three-state model best explained reaction time. Furthermore, the average log-likelihood of the Viterbi state restoration improved the fit quality of this explanatory model, indicating that SHMM confidence provided complementary behavioral information. These findings suggest that SHMMs trained only on fixation positions can identify phases broadly consistent with those traditionally observed in the decision-making process, such as early evidence accumulation and late decision confirmation.

## 1. Introduction

Hidden Markov chains [1] are commonly used in eye tracking to segment scan paths into homogeneous phases based on the statistical properties of eye movements. When fixation positions are used as observations, the hidden states correspond to regions of interest (ROIs) defined by recurrent fixation locations, while the transition matrices capture how these ROIs are visited over time [2,3].

Two previous studies [4,5] have demonstrated that Hidden Markov Models (HMMs) can account for inter-individual spatio-temporal variability during a face recognition task when applied to eye movements. Through unsupervised learning, these models have revealed two distinct classes of exploration profile among participants, depending on their cognitive style: holistic versus analytic [6]. However, these models assume that transition probabilities between ROIs remain stationary throughout the trial. This assumption may be challenged in complex tasks involving multiple successive cognitive phases, each of which is potentially associated with a distinct visual exploration pattern [7]. Indeed, research on decision-making has shown that eye movement behaviors evolve over time as participants gather, integrate, and verify relevant information for the decision [8,9,10]. In this context, it is worth considering that the different phases of a single task may be associated with distinct visual exploration dynamics.

To address this limitation, Switching Hidden Markov Models (SHMMs) enable the simultaneous modeling of low-level hidden states (i.e., ROIs) and higher-level hidden states (HIs), as proposed by Chuk and collaborators [2]. Although HI states share the same ROIs, they are characterized by distinct transition matrices. Therefore, SHMMs are particularly well-suited to modeling successive cognitive phase shifts within a single task.

Changes in cognitive state during a single trial or item arise in many paradigms. During perceptual or preference-based decision-making processes, a shift from evidence accumulation to consolidation is classically assumed and observed [11]. The following examples are illustrative rather than exhaustive. Chuk et al. [2] modeled such a shift in a face preference task based on ocular data. They showed a transition from an exploratory phase to a preference-biased period, which was linked to a gaze cascade effect [12]. In a problem-solving eye-tracking task, Wang and Zhan [13] identified within-item cognitive strategy switching (CSS) (i.e., switching from constructive matching to response elimination), in Raven matrices. In reading, Olivier et al. [14] detected a cognitive phase shift during a semantic decision task. Our task is similar to these studies in that shifts in cognitive states are also expected to occur during the decision-making process.

The most direct approach to segmenting a sequence into interpretable phases uses an explicit behavioral marker, such as a response or a reaction time, to delimit the segments prior to modeling. However, this trivial case is not always applicable: the marker may be unavailable, or deliberately excluded, as in our study. Excluding the marker from the model’s learning process keeps the segmentation fully unsupervised. Alternatively, the marker can be used afterward to evaluate the resulting segmentation. Thus, the key methodological question becomes how to segment sequences into interpretable phases without relying on such an explicit marker. Existing approaches differ in whether the experimental design provides spatially distinct areas that allow for a priori temporal segmentation into stationary segments. This is the case in the research by Wang and Zhan [13]. Their task presents two spatially separate areas (the response alternatives and the matrix), and each time the gaze toggles between these two areas, a new temporal segment—an “eye movement snippet”—begins. A conventional HMM can then be fitted to each temporal snippet separately. Other tasks like ours do not offer such a spatial marker. In these cases, segmentation must be estimated jointly with the transition dynamics, directly from fixation positions.

Three main approaches exist for this: change-point detection methods [15]; semi-Markov models, which explicitly model the distribution of state sojourn durations [16]; and switching models (SHMMs) [2], which model a distinct transition dynamic per high-level state. Change-point detection is a general signal processing technique that is widely used for fault detection and other applications. However, its application to eye movements for higher-level cognitive-phase segmentation remains challenging, notably with regard to the choice of a potentially multi-dimensional indicator. Beyond the limited number of transitions between cognitive phases (i.e., ruptures) per trial, this approach typically requires tuning decision thresholds, which is difficult to reconcile with the pronounced inter-trial and inter-participant variability that characterizes such cognitive tasks. To our knowledge, this approach has so far only been used for low-level segmentation of eye-movement profiles, such as smooth-pursuit onset and offset [17], rather than for cognitive-phase segmentation. A semi-Markov approach [16] was applied by Olivier and collaborators [14] to a text-reading task in which participants had to decide whether a previously presented theme was semantically related to the text’s content as they read. The model did not directly analyze fixation positions. Instead, it focused on transitions between fixations (saccades). To achieve this, a preprocessing step was applied, which associated each fixation with the closest word or group of words in the text. Then, a categorical variable representing the type of transition of each saccade (“backward” and “forward” with two different spans, and “re-fixation”) was used as the observed data. In this context, the semi-Markov model was based on variables observed beyond raw data (such as fixations or saccades), since these were enriched with information from the text’s words.

Building on this general framework of unsupervised model-based segmentation of cognitive phases, our study aimed to evaluate the ability of SHMMs to segment the scanpaths produced during a complex visual exploration and decision-making task. Inspired by the aeronautical domain, the experimental task involved monitoring the movement of an autonomous drone among obstacles simulating air traffic. Participants had to decide which direction the drone should move to best avoid obstacles. The relatively long duration of the presentation of successive obstacles allowed participants to go through multiple successive stages of the decision-making process, from gathering initial information to preparing and executing the response, and potentially verifying it.

The contribution of this study is therefore twofold. First, we tested whether SHMMs trained solely on fixation positions could recover a temporal segmentation consistent with these successive stages. Importantly, no prior annotation or reaction time was used for the model’s training. We applied this approach to a substantially more complex task than that on which SHMMs were originally validated (a face preference decision-making task [2]). This task combined several intertwined cognitive phases and pronounced inter-individual variability. Second, we characterized the resulting high-level states through a convergent set of behavioral and eye-movement indicators in relation to the decision-making process.

In summary, we chose to use SHMMs because they can identify both low-level states (i.e., ROIs) and high-level states (high-level features, HIs) through unsupervised learning without the need for any prior ROI classification. SHMMs were particularly well suited for our study because they can capture the complex temporal dynamics of scanpaths without requiring prior annotation, which is a challenging task in the context of complex decision-making. SHMMs also allow cognitive interpretation of the identified states [2]. Therefore, we used eye fixation positions as the model’s sole observations. The primary goal of this approach was to determine whether an SHMM could identify shifts in visual exploration dynamics corresponding to different phases of cognitive processing in the context of a complex visual exploration and decision-making task using data-driven segmentation, in a fully unsupervised manner.

Evidence-accumulation models are a theoretical framework that helps clarify the expected nature of these different phases. According to these models, information about the different alternatives is gradually integrated until the decision variable reaches a threshold that results in a response commitment [11]. When the relevant information is distributed throughout the visual environment, the accumulation depends on successive visual sampling [18,19]. The various pieces of information are fixated on successively. When the evidence is weak, the decision takes longer and involves more fixations and transitions between information sources [20,21]. The relationship between oculomotor exploration and accumulation has been formalized by attentionally modulated drift diffusion models. These models account for fixation sequences, choices, and reaction times based on fixated information [20,22,23]. Therefore, oculomotor exploration can be considered a component of the active process of evidence acquisition [24].

As the decision approaches, the exploratory dynamic gradually shifts toward a fixation on the chosen alternative. This choice is often reflected by a shorter fixation than previous ones [22,25]. This late-onset gaze bias [26] may reflect a post-decision process that precedes the observable motor response [27]. Once the response has been formulated, gaze may shift toward predictable future information [28,29]. This anticipatory orientation is particularly relevant to our task. Indeed, after selecting an avoidance trajectory, participants must verify the system’s decision, which appears in the central region of the screen during the next phase.

These studies allow us to predict the temporal organization of oculomotor behavior in our task. This organization should range from an initial phase of sampling information relevant to the decision, which is consistent with a process of evidence accumulation, to a later phase associated with committing to a response. This phase would be followed by an anticipatory reorientation toward the expected information in the next step. An SHMM does not directly model an evidence accumulation variable; therefore, its hidden states cannot be equated with cognitive accumulation states a priori. However, an SHMM allows us to test whether scanpaths exhibit a temporal segmentation consistent with this sequence of processes, through the unsupervised identification of successive periods characterized by distinct patterns of transitions between regions of interest (ROIs).

Given the exploratory nature of this study, we hypothesized that SHMMs with two or three high-level states could segment scanpaths into interpretable temporal phases despite substantial inter-individual variability. Specifically, we expected the identified states to align with the decision-making process and to exhibit distinct properties in terms of: (i) transition dynamics between ROIs, (ii) spatial distribution of fixations, and (iii) relationship with reaction times, even though the latter were not used during model training.

## 2. Materials

### 2.1. Participants

Fifty-six participants took part in the experiment (38 females, M = 22 years, SD = 4.2 years). Three participants were excluded from the sample due to a misunderstanding of the instructions or early termination. All participants had normal or corrected vision, no history of psychiatric or neurological disorders before or during the experiment, and were not taking any medication that could affect the nervous system. Participants provided written informed consent and were compensated with €90 for their participation. This research was approved by the Ethics Committee (Comité de Protection des Personnes Nord Ouest IV, reference no. 38RC24.0131).

### 2.2. Design

Participants completed three experimental sessions (Session 1: *Actor*; Sessions 2 and 3: *Supervisor*), spaced approximately nine days apart. While all three sessions were part of a larger project, this study only analyzed the eye-tracking and behavioral data from the first session (*Actor*), in which participants were required to make active decisions. The session included four blocks of 32 trials each, for a total duration of approximately 60 min. A 32-trial training phase (10 min) preceded it. A debriefing was conducted at the end of the final session, during which the experimental conditions and study objectives were explained. All sessions were performed at the IRMaGe Neurophysiology facility (Grenoble, France).

### 2.3. Procedure

The experimental task involved actively exploring a dynamic scene leading to a decision-making process. In an aeronautical supervision context, participants were required to supervise an autonomous drone that was always positioned at the center of a radar. Obstacles representing air traffic moved from the top to the bottom of the screen relative to the drone. The drone was equipped with an obstacle-avoidance system that was designed to detect obstacles and autonomously select one of two invariant avoidance trajectories: left or right. The system had a 25% error rate. During this session, similar to the drone’s control system, participants had to explore the scene containing moving obstacles and decide which direction to take to avoid them (left or right). They had to make this decision during the exploration phase. The chronological sequence of a trial is illustrated in Figure 1.

A trial began with an *Initialization* phase (2400 ms) during which no obstacles were presented. The following *Conflict* phase (6600 ms) involved the progressive appearance of obstacles from the top of the screen (see Section 2 for a description of the stimuli). During this phase, participants were instructed to indicate the avoidance trajectory the drone should follow (left or right) by pressing a response key as quickly as possible while minimizing errors.

During the *System Decision* phase (1000 ms), the system’s response was displayed in the center of the screen as a colored arrow. Then, the *Validation* phase began when the central circle around the drone turned blue; participants had to press a response key to validate the system’s decision if they deemed it correct.

Finally, the *Avoid* phase (14,500 ms) started when the central circle around the drone turned white. This phase simulated the drone’s movement along its chosen trajectory or the trajectory corrected by the participant. It thus allowed them to visualize the avoidance path among the obstacles. At the end of each trial, the participant was required to fixate on a circle appearing in the center of the screen. This allowed the experimenter to apply a drift correction or recalibration, if necessary.

A total of 128 trials were presented to participants, divided into four blocks of 32 trials each. For half of the trials (random order), the correct avoidance trajectory was to the left, and for the other half, it was to the right. Additionally, the system’s decision was incorrect in 25% of the trials (random order). Before the first session, participants completed a 32-trial training phase to learn the two possible symmetrical trajectories with respect to the vertical midline of the screen. The training trials followed the same temporal sequence of phases as previously described. Only the *Avoid* phase was replaced with:

- Feedback on the participant’s responses (2000 ms), concerning both the direction decision and validation;
- A visualization phase of the chosen trajectory (2500 ms), allowing participants to identify the positions of obstacles on or near the trajectory, i.e., those relevant to decision-making, for each trial.

Although eye movements and EEG activity were simultaneously recorded during the experiment, the present study only focused on the analysis of eye-tracking data. EEG data will be the subject of specific analyses in future work. For this study, we therefore analyzed only eye-tracking data (eye fixations) and behavioral data (decision to choose the avoidance trajectory: left or right) during the *Conflict* phase.

A photodiode placed on the screen detected a light appearing at the beginning of each of the five phases segmenting a trial. Upon each detection, a trigger was sent to the eye tracker via its parallel port to precisely measure the start and end of each phase.

The experiment was programmed in TypeScript and executed in a Node.js environment. Communication with the eye tracker was handled by a dedicated Python module, called from Node.js using the *node-calls-python* library (v1.10.0) [30]. Data exchange between the Node.js server, the eye tracker, and the graphical interface was implemented using the WebSocket protocol, via the *ws* library (v7.5.3) [31]. This was an adaptation of the program used by Salomone and collaborators [32].

### 2.4. Visual Stimuli

The visual scene consisted of:

- A drone represented in a central circle (radius 1°), fixed at the center of the screen;
- Obstacles, depicted as yellow triangular shapes within a circle (radius 0.45°), descending vertically from the top of the screen toward the drone;
- A neutral background, representing a stylized radar, with fixed speed and altitude information to avoid visual distractions.

The 18 obstacles were horizontally distributed: six on the left, six in the middle, and six on the right. The minimum distance between two adjacent obstacles was 2.2°. Their positions were updated at a frequency of 30 Hz.

During the trial, among the six obstacles located on the left or right of the drone’s trajectory, one, two or three reached their final positions, either on the trajectory or very close to it. For example, if these obstacles were on the right side, it would indicate that the left trajectory should be chosen to avoid them. These obstacles were relevant for determining whether the system’s avoidance decision (left or right) was correct. During the first 3600 ms, the obstacles appeared progressively and decelerated until reaching near-zero speed. At this moment, all obstacles were present and almost stationary. This configuration was maintained for the following 3000 ms, until the end of the *Conflict* phase. From the drone’s perspective, the change in obstacle speed simulated a deceleration as the drone approached them.

An example video (Trial.mp4) illustrating this scene for a trial is available in the repository referenced in the Data Availability Statement.

### 2.5. Apparatus

Each dynamic stimulus was displayed on a 27-inch screen with a resolution of 1920 × 1080 pixels and a refresh rate of 60 Hz. Participants were seated 60 cm from the screen. The stimuli covered a visual angle of 34.6° × 28.4°.

Eye position on the screen was recorded using an Eye-Link 1000 binocular infrared eye tracker (SR Research, Ottawa, ON, Canada) with a sampling rate of 1000 Hz. For each participant, the eye used for analysis was selected during the setup phase based on the pupil detection stability and the quality of the calibration-validation process. Calibrations were accepted when the average validation error was below 1° of visual angle and the maximum error was below 1.5°.

The Eyelink software (version EYELINK II 1) automatically detected saccades and fixations using three distinct thresholds: a minimum displacement of 0.1° from the previous eye position, a minimum velocity of 30°/s, and a minimum acceleration of 8000°/s^2^. A 9-point calibration was performed before the task and repeated during the blocks when necessary.

### 2.6. Behavioral Data

The behavioral data included the reaction time—the delay between the onset of the Conflict phase and the key press used to select the avoidance side (left or right).

### 2.7. Eye-Tracking Data

The start times, end times, and positions of eye fixations were obtained from the automatic extractions performed by the eye tracker. To prepare the SHMM learning based on eye fixation positions during the *Conflict* phase, each fixation position was classified into one of the following four categories:

- *Center* (within a 6° × 6° square at the center of the screen, area without obstacles and location of the system’s feedback during *System Decision* and *Validation* phases);
- *Bottom* (below a horizontal line extending across the full width of the screen and aligned with the lower edge of the previously described Center square, area without obstacles);
- *Out* (outside the screen);
- *Obstacle* (all other fixations).

Fixations in the *Obstacle* region contributed to task resolution because they were located in the part of the visual scene containing the moving obstacles. The *Center* region was particularly relevant to the task because the system’s choice for the avoidance side was displayed there. Fixations in the *Bottom* and *Out* regions were non-informative for task resolution, and did not contribute to SHMM learning.

## 3. Methods

### 3.1. Switching Hidden Markov Model

#### 3.1.1. Learning

Switching Hidden Markov Models (SHMMs) were used to model the participants’ eye movement behavior during the *Conflict* phase. To capture inter-individual variability, an SHMM was learned for each participant based on trials that:

- Had valid synchronization of photodiode markers;
- Were correctly solved by the participant;
- Contained more than three fixations.

This choice was consistent with the work of Chuk et al. [2], which showed that the transition dynamics between regions of interest differed primarily between eye movement patterns associated with correct and incorrect responses. Including both correct and incorrect trials could have introduced additional heterogeneity in the estimation of transition matrices. Among the selected trials, the first fixation, which was not informative for the task, as well as fixations classified as *Bottom* and *Out*, were excluded.

Since the obstacles moved from top to bottom and the task was to decide which side to avoid, the vertical position of the fixations was not considered. Therefore, the input data were one-dimensional, computed from the horizontal positions of the fixations. For trials in which the correct side was the right side (an arbitrary choice), these positions were transformed by mirror symmetry relative to the center of the screen. This transformation allowed all trials to be grouped together, by considering the correct side to always be the left. Additionally, to distinguish between fixations classified as *Center* and *Obstacle* after reduction to a single horizontal dimension, the horizontal positions of fixations classified as *Center* were offset by a constant to obtain negative values.

Two learning configurations were tested:

- R = 4 low-level states (denoted ROI, Regions Of Interest);
- S = 2 or S = 3 high-level states (HIs).

These two configurations were chosen a priori on theoretical grounds rather than through exploratory model selection over a broader range of states. This logic follows that of Chuk et al. [2], who fitted their SHMM to a face preference decision-making task with two pre-assumed cognitive states—an exploration period and a preference-biased period—rather than selecting the number of states based on data. Similarly, our two-state configuration directly reflects the minimal decision structure of the task (an exploratory phase followed by a decision phase), while the three-state configuration allows us to test whether a more fine-grained intermediate stage of the decision process can be identified. Our aim was not to determine the “optimal” number of HI states, but rather to assess whether SHMMs applied with a few theoretically motivated configurations could recover temporal segmentations consistent with the organization of the decision-making process. For completeness, a quantitative comparison between the two configurations is provided in Appendix A.

Hereinafter, the HI states were denoted by *HIn_S*, where *n* ranges from 1 to S and S = 2 or S = 3.

For each configuration, a participant-specific SHMM was learned based on their one-dimensional fixation positions. The initialization and configuration parameters were identical for all learning processes. They are summarized in Table 1.

These prior probabilities and initial transition matrices constrained the initial HI state to be the first (*HI1_2* or *HI1_3*), and the order of HI state visits was strictly increasing, with no possibility of returning to a previous state. Thus, at most one transition between adjacent HI states could occur during a trial. This allowed us to define a unique transition time between adjacent HI states for each trial. These transition times are denoted as follows:

- *THI12_2* for the SHMMs with S = 2,
- *THI12_3* and *THI23_3* for the SHMMs with S = 3.

This unidirectional transition structure (i.e., forward-only) was chosen to reflect the irreversible, cumulative nature of the studied decision-making process, in which participants progressively gather and consolidate evidence. This is consistent with the fact that participants were required to provide an explicit response, confirming that the experiment involved a genuine decision-making process leading to a choice rather than open-ended or reversible exploration. Unlike the study by Olivier et al. [14], whose semi-Markov model allowed less constrained transitions but was fitted jointly across all participants, our SHMMs were individually fitted to capture inter-individual variability in exploration strategies. Consequently, constraining the transition structure was necessary to keep the resulting HI states interpretable and to allow their meaningful correspondence across all individual models. This was facilitated by the standardization step described below (Section 3.1.2).

The SHMMs were implemented in MATLAB (R2023a, V9.14) using the toolbox (v0.1) developed by Chuk et al. [2]. After learning, the estimated parameters for each model were obtained:

- Prior probabilities and transition matrices for HI and ROI states;
- Parameters of Gaussian distributions of observations (means and variances) for ROI states;
- Global log-likelihood of the model.

#### 3.1.2. SHMM Standardized Representation and States Restoration

After learning the SHMMs, a standardization step was applied to ensure consistent naming of HI states and ROIs across participants. The HI states were numbered in descending order of their prior probabilities, and the ROIs were ordered in ascending order of their one-dimensional positions. After standardization, ROI 1 corresponded to the *Center* area (negative position), and the other three ROIs were ordered from left (correct avoidance side after mirror transformation) to right (incorrect side).

The Viterbi algorithm was then used to restore the combined ROI × HI sequence for each trial. For a given trial, this algorithm returns the most probable sequence of combined ROI × HI states given the observed fixation positions. The cumulative log-likelihood of restoration (Cml) corresponds to the joint log-likelihood of this restored sequence, cumulated over all fixations contributing to the trial (i.e., all fixations of the trial except for the first fixation and fixations classified as Bottom or Out, see Section 3.1.1). Since Cml is a sum over the fixations contributing to a trial, it scales approximately with the number of fixations. Therefore, trials with more fixations yield a more negative Cml value, regardless of segmentation quality. To obtain a confidence index of the SHMM-based segmentation quality that was not influenced by this mechanical dependency, we used the average log-likelihood of restoration (AvgLL). AvgLL is obtained by dividing Cml by the number of fixations contributing to the trial. We used AvgLL as our SHMM confidence index in subsequent analyses (see Section 3.3).

The consistency of the HI state standardization was verified by ensuring that the transition times between adjacent states were in ascending order.

Finally, experimental saliency maps were generated for each HI state using the two-dimensional positions of the original fixations [33,34]. The maps were obtained by convolution with a Gaussian kernel with a standard deviation of 2°.

### 3.2. Linear Mixed-Model Analyses of Participant-Level Measures

For analyses based on participant-level means, statistical inference was performed using Linear Mixed Models (LMMs). A random intercept for each participant was included in all models to account for the dependence among repeated observations from the same participant.

LMMs were implemented in MATLAB using the *fitlme()* function and estimated by maximum likelihood. Fixed effects were evaluated using marginal F-tests. Planned pairwise comparisons were performed by testing linear contrasts of the fitted model coefficients (*coefTest()* function) with Bonferroni correction for multiple comparisons. Effect sizes were quantified using partial eta-squared (η^2^p). Model assumptions were assessed by inspecting the random-effects variance and residuals of the final model, including their distribution, conditional independence, and homogeneity of variance.

### 3.3. Transition Times Between HI States

To evaluate the behavioral relevance of the segmentation produced by the SHMMs, we examined the relationship between the transition times of high-level states (HIs) and reaction times (RTs). Since RTs were not used during the learning phase, this analysis assessed whether the temporal segmentation estimated from fixation positions alone could explain the observed behavior, and thus confirm the interpretation of our HI states. No independent trial set was used for this assessment. Therefore, the analysis evaluated the association between the SHMM-derived transition times and RT within the same samples.

Three transition times were considered: the *THI12_2* transition from the two-state model (S = 2), as well as the *THI12_3* and *THI23_3* transitions from the three-state model (S = 3). Two complementary approaches were applied to each of these transitions.

First, the gap between the SHMM-estimated transition time and the observed RT was computed for each trial to evaluate the temporal proximity between the SHMM segmentation and the observed behavior directly. The absolute value of the gap was analyzed using LMMs with transition (*THI12_2*, *THI12_3*, and *THI23_3*) as a fixed effect.

Second, the relationship between RT and transition times was evaluated. Unlike the previous analyses, which were performed on participant-level means, the relationship between transition times and RT was analyzed at the trial level using LMMs. For each transition time, we compared three models of increasing complexity:

- A fixed-effects-only model;
- A model including a random intercept per subject;
- A model including both a random intercept and a random slope per subject.

The models were compared using the Akaike Information Criterion (AIC) and log-likelihood. The model with the best trade-off between fit quality and parsimony was retained for each transition time. According to Nakagawa and Schielzeth [35], model fit was further quantified using the marginal R^2^ (variance explained by the fixed effects alone) and the conditional R^2^ (variance explained by the fixed effects together with the random effects). Since the conditional R^2^ combines the contributions of the fixed effects and random effects—including stable between-participant differences in RT captured by the random intercept and/or slope—it should not be interpreted as reflecting the specific explanatory contribution of a given fixed effect. Therefore, marginal R^2^ was only compared between models sharing the same random-effects structure, since both marginal and conditional R^2^ depend on this structure.

Finally, the average log-likelihood of the Viterbi state restoration (AvgLL) was added as an additional explanatory variable to evaluate whether the confidence of the SHMM segmentation provided complementary information for explaining RT.

The tested hypotheses were as follows:

1. If the transitions detected by the SHMM are linked to RTs, then it suggests that HI states reflect the relevant behavioral stages of the decision-making process.
2. If the AvgLL variable improves the explanation of RTs, then it suggests the trials that are better described by the SHMM exhibit a more consistent temporal organization.

### 3.4. Cumulative Dwell Times During States

To interpret the HI states of the SHMMs, the cumulative dwell times in the different ROIs were computed for each HI state. For a given HI state and ROI, the cumulative dwell time corresponded to the sum of fixation durations assigned to that HI state and ROI. Then, the mean cumulative dwell times across trials were computed for each participant and analyzed using LMMs with HI state, ROI, and their interaction as fixed effects.

### 3.5. Kullback–Leibler Divergence

The Kullback–Leibler (KL) divergence was used for two purposes:

1. To characterize the transitional state *HI2_3* from the SHMM with three HI states. This is an intermediate state between *HI1_3* and *HI3_3*.
2. To assess inter-individual variability in the HI state representations.

For each SHMM, each HI state was characterized by a 4 × 4 transition matrix (R = 4), which reflected the transition probabilities between ROIs (low-level states) during that HI state. The KL divergence, which measures the dissimilarity between probability distributions, was used here to quantify the differences between these matrices. Specifically, the KL divergence *D_KL_*(*P*‖*Q*) was computed separately for each row of two transition matrices (P, Q), with each row representing a probability distribution over the four ROIs (sum to one). A small epsilon value (1.10^−10^) was added to each probability to avoid undefined logarithms when probabilities were zero, followed by renormalization of each row. The four resulting row-wise KL divergences were then averaged to obtain a single KL divergence value *D_KL_*(*P*‖*Q*) for each comparison. The KL divergences were transformed using a Box–Cox transformation [36] with a common λ parameter to approximate a normal distribution across all distributions. The λ parameter was then determined to optimize global normality across all conditions.

#### 3.5.1. Intermediate State HI2_3 Obtained from SHMMs with 3 HI States

To characterize the role of the intermediate state *HI2_3* in the exploration dynamics, we compared its transition matrix with those of the other two states obtained from SHMMs with three HI states, namely *HI1_3* and *HI3_3*, using KL divergence. For each participant, the KL divergence *D_KL_*(*P*‖*Q*) was computed, where P was the transition matrix of the comparison state (*HI1_3* or *HI3_3*) and Q was the transition matrix of the reference state *HI2_3*. The four row-wise divergences were averaged to obtain a single KL-divergence value for each comparison (*D_KL_*(*HI1_3*‖*HI2_3*) or *D_KL_*(*HI3_3*‖*HI2_3*)). Comparing these KL divergences allowed us to determine whether these transition dynamics of the intermediate state were more similar to the dynamics of evidence accumulation (*HI1_3*) or confirmatory exploration (*HI3_3*). After a Box–Cox transformation, KL Divergence between *HI2_3* and *HI1_3*, and between *HI2_3* and *HI3_3* were analyzed using LMMs with HI state (*HI1_3*, *HI3_3*) as a fixed effect.

The same methodology was applied to compare the intermediate state *HI2_3* with the two states *HI1_2* and *HI2_2* obtained from SHMMs with two HI states.

#### 3.5.2. Variability of Transition Matrices

Interindividual variability of HI states was assessed by analyzing the transition matrices between ROIs for each of the five HI states (*HI1_2*, *HI2_2*, *HI1_3*, *HI2_3*, and *HI3_3*), which were obtained from individual SHMMs with two and three HI states. For each HI state, the KL divergence was computed between the transition matrix of that HI state for each participant and the average transition matrix for that HI state across all participants. For each participant and HI state, the individual SHMM’s transition matrix was used as P, and the corresponding average transition matrix across participants was used as Q. This yielded *D_KL_*(P‖*Q*). Thus, this divergence quantified the difference between the individual representation and the average representation of the HI state. This approach allowed us to quantify the inter-individual variability in how individual SHMMs characterized each HI state.

To analyze the variability of HI state representations, informed by KL divergences, we used LMMs after applying a Box–Cox transformation.

The transformed KL divergences were analyzed using LMMs with HI state (*HI1_2*, *HI2_2*, *HI1_3*, *HI2_3*, and *HI3_3*) as a fixed effect.

## 4. Results

### 4.1. Number of Trials and Fixations and Reaction Time

Participants correctly solved 92.9% of the trials (SE = 1.27). After applying the inclusion criteria, an average of 117.5 trials per participant (SE = 1.68) was retained for each SHMM learning. Retained trials contained an average number of 16.4 fixations (SE = 0.33), of which an average of 14.9 fixations (SE = 0.33) contributed to SHMM learning.

The mean reaction time (RT) was 3192.4 ms (SE = 135.59).

### 4.2. Experimental Saliency Maps for Each HI State

The experimental saliency maps for each HI state are illustrated in Figure 2. After standardization, the four ROIs were ordered by increasing 1D position: ROI 1 to ROI 4. For interpretability, they were renamed as follows: ROI 1 = *Center* (negative 1D position, corresponding to the drone’s location), ROI 2 = *ValidSide* (left, the correct avoidance side after the mirror transformation), ROI 3 = *Middle* (the intermediate area between *ValidSide* and *WrongSide*), and ROI 4 = *WrongSide* (right, the incorrect avoidance side after the mirror transformation).

Based on these maps, several observations were drawn:

- In the early states (*HI1_2*, *HI1_3*, and *HI2_3*), exploration primarily occurred in the *ValidSide*, *Middle*, and *WrongSide* ROIs. Moreover, participants fixated on the *ValidSide* ROI more often.
- During the later states (*HI2_2* and *HI3_3*), exploration primarily occurred in the *Center* ROI, while exploration between the *ValidSide* and *WrongSide* ROIs continued.

To validate these spatial patterns, statistical analyses of cumulative dwell times across HI states and ROIs were conducted, as described in the following section.

### 4.3. Characterization of HI States by Cumulative Dwell Times in Low-Level States (ROI)

An LMM was used to analyze the effect of the high-level states (HIs) and the low-level states (ROI) on the cumulative dwell time. For both model configurations (S = 2 and S = 3), the variance of the random intercept for each participant was estimated at the boundary of the parameter space (≈0 in both cases). This indicates that once the fixed effects (HI, ROI, and their interaction) were included, no participant-level variance remained unaccounted for. Since the fixed-effects-only model is numerically equivalent to the random-intercept model in this case, it is reported for the subsequent analyses.

#### 4.3.1. SHMM with Two HI States

The main effect of the HI state (HI: *HI1_2*, *HI2_2*) was significant (F(1, 415) = 273.49, η^2^p = 0.40, *p* < 0.0001), with a longer cumulative dwell time in *HI2_2* (1222.20 ± 30.64 ms) than in *HI1_2* (879.70 ± 32.61 ms) (Figure 3a).

The main effect of the low-level state (ROI: *Center*, *Middle*, *ValidSide*, *WrongSide*) was also significant (F(3, 415) = 18.43, η^2^p = 0.12, *p* < 0.0001) (Figure 3b). Post-hoc comparisons with Bonferroni correction revealed that the cumulative dwell time in the *Center* ROI was significantly longer (1309.67 ± 74.50 ms) than in the *ValidSide* ROI (1195.83 ± 36.77 ms, F(1, 415) = 55.01, *p* < 0.0001), the *Middle* ROI (837.20 ± 42.09 ms, F(1, 415) = 16.40, *p* < 0.001), and the *WrongSide* ROI (856.91 ± 24.73 ms, F(1, 415) = 16.86, *p* < 0.001). Notably, the cumulative dwell time in the *ValidSide* ROI (1195.83 ± 36.77 ms) was significantly longer than in the *Middle* ROI (837.20 ± 42.09 ms, F(1, 415) = 11.34, *p* < 0.01) and the *WrongSide* ROI (856.91 ± 24.73 ms, F(1, 415) = 10.96, *p* < 0.01).

The HI × ROI interaction was significant (F(3, 415) = 76.02, η^2^p = 0.35, *p* < 0.0001), indicating that the effect of the low-level state (ROI) on the cumulative dwell time depended on the HI state (Figure 3c). Specifically, in *HI1_2*, the cumulative dwell time in the *Center* ROI (497.17 ± 104.43 ms) was significantly shorter than in the *ValidSide* ROI (1226.00 ± 80.06 ms, F(1, 415) = 55.01, *p* < 0.0001), the *Middle ROI* (895.12 ± 70.41 ms, F(1, 415) = 16.40, *p* < 0.001), and the *WrongSide* ROI (900.64 ± 41.78 ms, F(1, 415) = 16.86, *p* < 0.001). In contrast, in *HI2_2*, the cumulative dwell time in the *Center* ROI (2122.20 ± 90.16 ms) was significantly longer than in the *ValidSide* ROI (1165.70 ± 53.22 ms, F(1, 415) = 147.07, *p* < 0.0001), the *Middle* ROI (777.11 ± 56.45 ms, F(1, 415) = 156.57, *p* < 0.0001), and the *WrongSide* ROI (813.18 ± 34.17 ms, F(1, 415) = 151.86, *p* < 0.0001). Notably, while the cumulative dwell time in the *ValidSide* ROI (1226.00 ± 80.06) was significantly longer than in the *WrongSide* ROI during *HI1_2* (900.64 ± 41.78 ms, F(1, 415) = 10.96, *p* < 0.05), these two times did not differ during *HI2_2* (1165.70 ± 53.22 ms vs. 813.18 ± 34.17 ms, *p* = 1.00). The interaction effect was primarily driven by the *Center* ROI, for which the cumulative dwell time during *HI2_2* (2122.20 ± 90.16 ms) was significantly longer than during *HI1_2* (497.17 ± 104.43 ms, F(1, 415) = 273.49, *p* < 0.0001).

#### 4.3.2. SHMM with Three HI States

The main effect of the high-level state (HI: *HI1_3*, *HI2_3*, *HI3_3*) was significant (F(2, 602) = 233.30, η^2^p = 0.44, *p* < 0.0001) (Figure 4a). Post-hoc comparisons with Bonferroni correction showed that the cumulative dwell time in *HI3_3* (1150.62 ± 35.62) was significantly longer than in *HI2_3* (690.97 ± 48.76 ms, F(1, 602) = 281.6, *p* < 0.0001) and *HI1_3* (658.34 ± 32.08 ms, F(1, 602) = 281.6, *p* < 0.0001). Additionally, the cumulative dwell time in *HI1_3* (658.34 ± 32.08 ms) was significantly shorter than in *HI2_3* (690.97 ± 48.76 ms, F(1, 602) = 5.8845, *p* < 0.05).

The main effect of the low-level state (ROI: *Center*, *Middle*, *ValidSide*, *WrongSide*) was also significant (F(3, 602) = 11.02, η^2^p = 0.05, *p* < 0.0001) (Figure 4b). Post-hoc comparisons with Bonferroni correction showed that the cumulative dwell time in the *Center* ROI was significantly longer (1059.11 ± 59.00 ms) than in the *ValidSide* ROI (919.58 ± 32.39 ms, F(1, 602) = 26.64, *p* < 0.0001), the *Middle* ROI (678.09 ± 28.48 ms, F(1, 602) = 22.83, *p* < 0.0001), and the *WrongSide* ROI (691.86 ± 21.18 ms, F(1, 602) = 12.24, *p* < 0.01).

The HI × ROI interaction was significant (F(6, 602) = 55.67, η^2^p = 0.36, *p* < 0.0001), indicating that the effect of the low-level state (ROI) on the cumulative dwell time depended on the high-level state (Figure 4c). Specifically, in *HI1_3*, the cumulative dwell time in the *Center* ROI (359.42 ± 44.69 ms) was significantly shorter than in the *ValidSide* ROI (818.97 ± 72.27 ms, F(1, 602) = 26.64, *p* < 0.0001), the *Middle* ROI (782.90 ± 54.76 ms, F(1, 602) = 22.83, *p* < 0.0001), and the *WrongSide* ROI (669.48 ± 42.72 ms, F(1, 602) = 12.24, *p* < 0.05). In *HI2_3*, the cumulative dwell time in the *ValidSide* ROI (934.38 ± 69.30 ms) was significantly longer than in the *Center* ROI (583.66 ± 112.03 ms, F(1, 602) = 14.00, *p* < 0.01) and *Middle* ROI (519.05 ± 59.73 ms, F(1, 602) = 21.11, *p* < 0.001), while the dwell time in the *ValidSide* ROI (934.38 ± 69.30 ms) did not differ from that in the *WrongSide* ROI (693.71 ± 39.39 ms, *p* = 0.25). Finally, in *HI3_3*, the cumulative dwell time in the *Center* ROI (2128.10 ± 92.34 ms) was significantly longer than in the *ValidSide* ROI (985.00 ± 53.01 ms, F(1, 602) = 167.97, *p* < 0.0001), the *Middle* ROI (735.40 ± 42.16 ms, F(1, 602) = 249.34, *p* < 0.0001), and the *WrongSide* ROI (693.84 ± 30.90 ms, F(1, 602) = 251.35, *p* < 0.0001). Notably, in *HI3_3*, the cumulative dwell time in the *ValidSide* ROI (985.00 ± 53.01 ms) was significantly longer than in the *WrongSide* ROI (693.84 ± 30.90 ms, F(1, 602) = 10.36, *p* < 0.05). As with the two-state SHMM, the interaction effect was primarily driven by the *Center* ROI, for which the cumulative dwell time during *HI3_3* (2128.10 ± 92.34 ms) was significantly longer than in *HI1_3* (359.42 ± 44.69 ms, F(1, 602) = 398.31, *p* < 0.0001) and *HI2_3* (583.66 ± 112.03 ms, F(1, 602) = 281.60, *p* < 0.0001).

### 4.4. Interpretation of the HI2_3 State from the SHMM with Three HI States

The *HI2_3* state obtained from the SHMM with three HI states was a transitional state between the first state (*HI1_3*) and the last state (*HI3_3*). Individual KL divergences were computed between the transition matrix of this transitional state (*HI2_3*) and those of the *HI1_3* and *HI3_3* states. These KL divergences were transformed using a Box–Cox transformation (λ = 0.5) to approximate a normal distribution (Shapiro–Wilk test, *p* > 0.05 for all states). These KL divergences were then compared using a random-intercept LMM. The results showed that the KL divergence between *HI2_3* and *HI1_3* (6.55 ± 0.54) was significantly lower than the KL divergence between *HI2_3* and *HI3_3* (7.39 ± 0.48; F(1, 104) = 16.13, η^2^p = 0.13, *p* < 0.001). The same methodology was applied to the two HI states, *HI1_2* and *HI2_2*, obtained from the SHMM with two HI states. The results showed that the KL divergence between *HI2_3* and *HI1_2* (4.54 ± 0.52) was significantly lower than the KL divergence between *HI2_3* and *HI2_2* (6.21 ± 0.48, F(1, 104) = 64.79, η^2^p = 0.38, *p* < 0.0001).

These results consistently suggest that the exploration dynamics during this transitional *HI2_3* state were more similar to evidence accumulation dynamics than to confirmatory exploration dynamics.

### 4.5. Inter-Individual Variability of HI State Characterizations

Each HI state was characterized by a transition matrix between the four ROIs (R = 4). To assess the inter-individual variability of this HI state characterization, KL divergences were calculated between the individual transition matrix (of each participant) for a given HI state and the average transition matrix for that HI state across all participants. A high KL divergence value indicated that an individual’s representation was far from the group average, thus reflecting high inter-individual variability. The KL divergences were transformed using a Box–Cox transformation (λ = −0.5) to approximate a normal distribution (Shapiro–Wilk test, *p* > 0.05 for all states). These KL divergences were then compared using a random-intercept LMM. The results showed a significant effect of the HI state (F(4, 260) = 14.81, η^2^p = 0.18, *p* < 0.0001). Post-hoc comparisons with Bonferroni correction revealed two state groups characterized by a significant inter-individual variability difference (Table 2):

- Group 1 (final states): Low inter-individual variability states, *HI2_2* and *HI3_3*, without significant inter-individual variability difference between these states.
- Group 2 (early states): High inter-individual variability states, *HI1_2*, *HI1_3*, and *HI2_3*, without significant inter-individual variability difference between these states.

These results suggest that the final states (*HI2_2* and *HI3_3*) were more homogeneous across individuals, whereas the early states (*HI1_2*, *HI1_3*, and *HI2_3*) exhibited greater variability, which may be related to differences in individual exploration strategies.

### 4.6. Analysis of Transition Times Between HI States

#### 4.6.1. Gap Between Transition Times and Reaction Time (RT)

To assess the temporal accuracy of the segmentations estimated by the SHMMs, the difference between the SHMM-estimated transition time and the observed reaction time (RT) was calculated for all participants and trials. A negative gap indicated that the estimated transition occurred earlier than the RT, while a positive gap indicated a later transition. This analysis was conducted for the three transitions between HI states: *THI12_2* (S = 2), *THI12_3*, and *THI23_3* (S = 3). Descriptive statistics across all trials and participants are presented in Table 3. The gap distributions for the three transitions exhibited high standard deviations (between 1442 ms and 1711 ms), reflecting substantial variability across trials and participants. The skewness coefficients were close to 0 (between −0.21 and 0.04), suggesting approximately symmetric distributions, while the kurtosis coefficients were close to 3 (between 2.99 and 3.30), indicating distributions close to normality.

Statistical analyses were performed on the individual means of the absolute gap values per participant for each transition, to compare differences between transition types at the group level (Figure 5).

A random-intercept LMM was used with the absolute gap as the dependent variable and the type of transition (*THI12_2*, *THI12_3*, and *THI23_3*) as fixed effect. The effect of transition type on the absolute gap was significant (F(2, 156) = 8.31, *p* < 0.001). Post-hoc comparisons with Bonferroni correction showed that the absolute gap was significantly smaller for *THI23_3* (1215.40 ± 56.46 ms) than for *THI12_2* (1398.68 ± 70.69 ms, F(1, 156) = 5.98, *p* < 0.05) and *THI12_3* (1518.90 ± 84.40 ms, F(1, 156) = 16.39, *p* < 0.001). No significant difference was observed between *THI12_2* and *THI12_3* (F(1, 156) = 2.57, *p* = 0.3324).

The estimated *THI23_3* transitions were significantly closer to the observed RTs than the other two transitions, suggesting better temporal precision for this transition in the SHMM with three HI states.

#### 4.6.2. Statistical Fit of Reaction Time to Transition Times

Reaction time (RT) was separately fitted from each of the three transition times using LMMs of increasing complexity. The results are presented in Table 4.

Using each of the three transition times as the sole explanatory variable, the LMM including a random intercept and a random slope per participant showed the best compromise between fit quality and parsimony (lowest AIC and highest R^2^). Consistent with the previous analysis, the THI23_3 transition best explained RT compared to the other two transitions, with the highest marginal R^2^ (7.22%) among the three transitions (2.12% for THI12_2 and 3.09% for THI12_3). This relative ranking was preserved across random-effects structures, confirming that the transition time to the final HI state (HI3_3) was the variable most strongly associated with RT.

Introducing a random intercept greatly improved the model fit (substantial increase in the conditional R^2^), revealing substantial inter-individual variability in baseline RT levels, with some participants consistently responding faster than others. Adding a random slope provided a more modest improvement, suggesting that individual differences are more related to global decision speed than to the relationship between transition time and RT.

For THI23_3, the model including AvgLL_3 as an additional explanatory variable could not be reliably estimated when both a random intercept and a random slope were included, due to a near-singular covariance matrix. We therefore retained the simpler random-intercept model for this analysis. Adding AvgLL_3 to this model improved its fit (in bold in Table 4): the marginal R^2^ increased from 2.97% to 3.17% (a modest relative increase of 6.7%), the AIC decreased by 35, and the residual standard deviation decreased by 2.6 ms. The coefficient β_2_ associated with AvgLL_3 for this model was −173.76 ± 28.48 (*p* < 0.0001), indicating that trials with higher SHMM confidence had lower RTs.

These results showed that the transition times estimated by the SHMMs explained a modest yet significant portion of the RT variance at the fixed-effects level (marginal R^2^), with a conditional R^2^ reaching up to 62.82% for the THI23_3 transition. As mentioned in Section 3.3, this conditional value represents the joint contribution of transition time and stable between-participant differences in RT, rather than the specific explanatory contribution of the THI23_3 transition itself.

Finally, the confidence variable AvgLL provided a small, yet statistically significant amount of complementary information. This improved the quality of the fit, independently of the number of fixations contributing to each trial.

## 5. Discussion

This study aimed to demonstrate how a Switching Hidden Markov Model (SHMM) can achieve a relevant spatiotemporal segmentation of a complex experimental task involving multiple cognitive processes and substantial interindividual variability. This expands the scope of the SHMM framework beyond its original application to face preference decision-making tasks [2], to include a dynamic task combining several intertwined cognitive phases and significant inter-individual variability. The experimental task was contextualized in the aeronautical domain and involved monitoring the movement of an autonomous drone navigating obstacles simulating air traffic and gradually descending toward the drone. Based on their exploration of the obstacle configuration in each trial, participants had to decide as quickly as possible which side (left or right) the drone should take to avoid the obstacles while minimizing errors. Obstacles appeared progressively during each trial, allowing participants to build their decision incrementally as they explored the scene. This setup enabled them to verify information before responding and continue confirmatory exploration after providing their decision.

Eye fixation positions were used as observations to identify different spatio-temporal patterns of visual exploration. In this exploratory approach, we hypothesized that SHMMs with two or three HI states could segment scanpaths into interpretable cognitive phases that occur at time points consistent with reaction times, even though reaction times were not used during training. The results supported this hypothesis, as both the two- and three-HI-state SHMMs produced segmentations that were consistent with the temporal organization of the decision-making process. Notably, the analysis of reaction times suggested the existence of cognitive phases of comparable duration, with an average reaction time of 3192.4 ms within the 6600-ms obstacle presentation phase constituting the *Conflict* Phase.

Unlike Chuk et al. [2], who trained separate SHMMs based on target lateralization, we applied a mirror transformation to the fixation positions relative to the vertical central axis of the screen. This allowed us to pool all trials into a single representational space, defining the left side as the correct side for all trials. This strategy normalized lateralization without altering the relevant cognitive information, and increased the effective sample size available for SHMM training. It also simplified the interpretation of low-level states because the *ValidSide* ROI always corresponded to the correct side and the *WrongSide* ROI always corresponded to the incorrect side after transformation.

The choice of a two-HI-state configuration was consistent with the task structure, which involved an explicit decision-making process during visual exploration. Due to the substantial inter-individual variability associated with this type of task, we also tested a three-HI-state configuration to determine whether the model would identify a more ambiguous intermediate stage. The HI states can be interpreted as successive stages of the decision-making process, ranging from an early phase dominated by information search to a later phase of decision verification and consolidation [11]. This interpretation aligns with the model structure, in which transitions are constrained to follow a forward temporal order, with no return to a previous HI state. This constraint was particularly well-suited to the task, during which relevant information appeared progressively, allowing participants to gradually consolidate their decision as they accumulated evidence.

The high accuracy rate (92.9%) demonstrated that participants reliably performed the task, which further supports the interpretation that HI states reflect effective decision-making processes.

Analyzing the inter-individual variability in HI states provided additional support for interpreting them as successive phases of the decision-making process. Calculating the Kullback–Leibler divergences between individual transition matrices and the average transition matrix for each state revealed two distinct groups: the final states of both models (*HI2_2* and *HI3_3*) exhibited low inter-individual variability, whereas the early states (*HI1_2*, *HI1_3*, and *HI2_3*) were significantly more variable. These results suggest that participants employed more diverse visual strategies during the initial phases of information gathering and integration [8], before converging toward more homogeneous behaviors as the decision emerged [9,16]. For the three-state HI model, this interpretation was further supported by the analysis of the intermediate state (*HI2_3*), whose dynamics appear closer to those of the early HI states than the final HI states. Thus, this intermediate state seems to correspond to an advanced stage of evidence accumulation rather than a distinct phase of decision consolidation. Within the framework of sequential decision-making models [11], these findings suggest that the three-state HI model may not reveal a third independent cognitive process, but rather it could provide a finer decomposition of the pre-decisional phase preceding commitment to a response. This interpretation aligns with the temporal analyses presented below, which point to the transition to *HI3_3* as the event most closely associated with reaction time.

Analyzing saliency maps and dwell times in the ROIs (low-level states) provided additional support for the cognitive interpretation of HI states. The experimental saliency maps revealed that the early states (*HI1_2*, *HI1_3*, and *HI2_3*) were primarily focused on the lateralized ROIs (*ValidSide* and *WrongSide*), where obstacles approaching the drone’s potential trajectory were displayed. In contrast, the final HI states (*HI2_2* and *HI3_3*) were characterized by a strong attraction to the *Center* ROI. Despite the inter-individual variability observed in the early HI states, the dwell time analysis revealed that participants spent more total fixation time within the *ValidSide* ROI than the *WrongSide* ROI, particularly in the HI1_2 state. Since only correctly resolved trials were used for SHMM training, this result suggests a progressive accumulation of evidence in favor of the upcoming correct response [11]. In the three-state HI model, this over-fixation of the *ValidSide* ROI relative to the *WrongSide* ROI did not reach statistical significance, likely due to the subdivision of the early phase into two separate states. However, in the *HI3_3* state, the threshold for significance was reached, suggesting that this over-fixation could correspond to a verification or confirmation activity of the decision. Finally, the final states of both models (*HI2_2* and *HI3_3*) were characterized by a significant increase in fixations on the *Center* ROI. This change is particularly informative because the central region did not contain obstacle-related evidence during the *Conflict* phase and was therefore of limited relevance for selecting the avoidance trajectory. However, participants knew from the repeated task structure that the system’s decision would subsequently be presented at this invariant central location during the *System Decision* phase. Thus, the increased attraction toward the *Center* ROI may reflect a transition from evidence sampling to anticipatory orienting toward the location of the next task-relevant event. Such an interpretation aligns with studies showing that gaze can be proactively shifted toward the expected location of an upcoming visual event, particularly when its timing and location are predictable [28,29]. Pfeuffer et al. demonstrated that gaze shifts toward an expected event location prior to its occurrence [28]. For their part, Drążyk and Missal found that such anticipatory saccades increase as the certainty about the target’s future location grows [29]. Thus, because the location of the system feedback was fully predictable in the present task, the central fixation pattern observed in the final HI states is consistent with an anticipatory reallocation of visual attention toward information required for the next stage of the task rather than continued sampling of evidence relevant to the current decision.

One of the key findings of this study is the observed relationship between the transition times of HI states and the participants’ reaction times, particularly since the latter were not used during SHMM training. Firstly, the gaps between the transition times of two adjacent HI states and the reaction times were calculated for each transition configuration (*THI12_2*, *THI12_3*, and *THI23_3*). Regardless of the transition time considered, the high variability in these differences (standard deviation greater than 1443 ms) indicates that the temporal alignment greatly varied across trials. In some trials, the SHMM transition occurred well before the behavioral response (highly negative gap), whereas in others, it occurred after the response (positive gap). Of the three transitions studied, *THI23_3* emerged as the most informative. Indeed, this transition was significantly closer to the observed reaction time than *THI12_2* and *THI12_3*. Secondly, reaction times were fitted using an LMM with each transition time configuration. Using maximum likelihood estimation, the model including the *THI23_3* transition provided the best statistical fit to reaction time, with a conditional R^2^ reaching up to 62.8%. These results are consistent with previous analyses showing that *HI1_3* and *HI2_3* shared similar properties regarding inter-individual variability and exploration dynamics, whereas *HI3_3* appears to be a qualitatively different HI state. Thus, the *THI23_3* transition may correspond to a pivotal moment in the decision-making process, marking the shift from a pre-decisional phase of progressive evidence accumulation to a later phase involving decision consolidation, verification, or preparation for the next stage of the task. This suggests that the transitions detected by the SHMM reflect not only statistical changes in scanpaths, but may also correspond to meaningful cognitive stages of the decision-making process. Furthermore, adding the average log-likelihood of state restoration (AvgLL_3) for the three-state HI model as an additional explanatory variable improved the fit of the reaction time model, with a modest increase in the marginal R^2^ (from 2.97% to 3.17%) and a corresponding reduction in the AIC. The average log-likelihood (AvgLL_3), which is derived from the Viterbi algorithm used for state restoration in the three-state HI SHMM, was used as a model confidence index to provide a more sensitive characterization of behavioral transitions between the different HI phases of visual scene exploration. Thus, trials associated with higher SHMM confidence were characterized by shorter reaction times. This result suggests that the quality of the segmentation estimated by the SHMM itself constitutes relevant behavioral information, albeit to a modest extent. Eye movement sequences that were more consistent with the model’s learned structure appear to be associated with faster decisions, whereas more ambiguous sequences were associated with longer decision times. Finally, the major improvement brought by introducing random intercepts in the LMMs highlights the importance of inter-individual variability in this type of task. While the relationship between SHMM transitions and reaction time was robust at the group level, individual differences in overall decision speed were still a significant source of variance. This result is consistent with previous analyses showing that the early phases of the decision-making process are precisely those during which visual strategies diverged the most between participants.

As outlined in the introduction, the correspondence between HI states and the specific cognitive constructs, such as evidence accumulation, verification or consolidation, was derived from the convergence of several exploratory indicators. These indicators include spatial distribution, dwell times, interindividual variability and the relationship with reaction time. This interpretative proposal requires confirmation through replication and in-depth analysis drawing jointly on neurophysiological brain data.

This study had two main limitations that should be highlighted. Firstly, HI states are latent variables inferred from eye movements. Although the model revealed transitions that were coherent with fixation dynamics and significantly related to reaction times, it did not permit direct observation of the underlying cognitive processes. Therefore, interpreting these states in terms of evidence accumulation, verification, or decision consolidation remains indirect. Secondly, while the constraint that prohibits backward transitions is suitable for tasks involving progressive decision-making, it limits the model’s ability to represent more flexible behaviors involving successive reevaluations or alternations between different exploration modes.

A first perspective for future research would be to strengthen the cognitive validation of HI states. Although the present study interpreted them based on the convergence of behavioral and eye-tracking indicators, the simultaneous recording of EEG activity in our study offers the possibility of investigating the neurophysiological correlates associated with transitions between states. Therefore, the SHMM could serve as a temporal segmentation framework for analyzing fixation-related event potentials or other neural markers involved in decision-making. A second perspective would be to characterize visual strategies within HI states more finely. Early and late states may differ, for example, in their balance of exploratory and exploitative behaviors [37,38]. Metrics such as saccade amplitude, velocity, or vigor could provide a more detailed description of these oculomotor dynamics [39]. A third perspective concerns the generalization of the participant-specific SHMMs. Future work should assess whether models learned from a subset of trials can reproduce a similar HI-state organization and transition timing in unseen trials from the same participant. In addition, comparing correct and incorrect trials would also help determine whether the identified temporal organization is specifically associated with successful decision formation. However, the low proportion of incorrect responses in the present experiment (7.1%) limited such an analysis at the individual level. Generalization could also be assessed across task contexts. Participants also completed two sessions in a strict supervisory role, during which no explicit response was required. Preliminary analyses suggest that the SHMMs obtained in this *Supervisor* condition are organized similarly to those observed in the *Actor* condition analyzed here. Comparing these two contexts could help assess the extent to which HI states reflect general decision-making processes, independent of the explicit expression of the decision.

In conclusion, this study shows that an SHMM trained only on eye fixation positions can identify temporal phases that align with the organization of the decision-making process. Specifically, converging results from Kullback–Leibler divergences, saliency maps, dwell times and reaction times are consistent with the cognitive labels associated with these states, which we had initially hypothesized based on existing literature regarding decision-making processes. However, replication studies or studies incorporating neurophysiological brain data could further substantiate our interpretation. These findings offer new perspectives on studying complex ecological tasks that are characterized by high inter-individual variability, and for which the explicit temporal segmentation of cognitive processes remains challenging. Beyond merely describing scanpaths, the identified high-level states appear closely linked to decision dynamics and provide a promising framework for jointly studying eye movements, behavior, and brain activity.

## Figures and Tables

**Figure 1:**
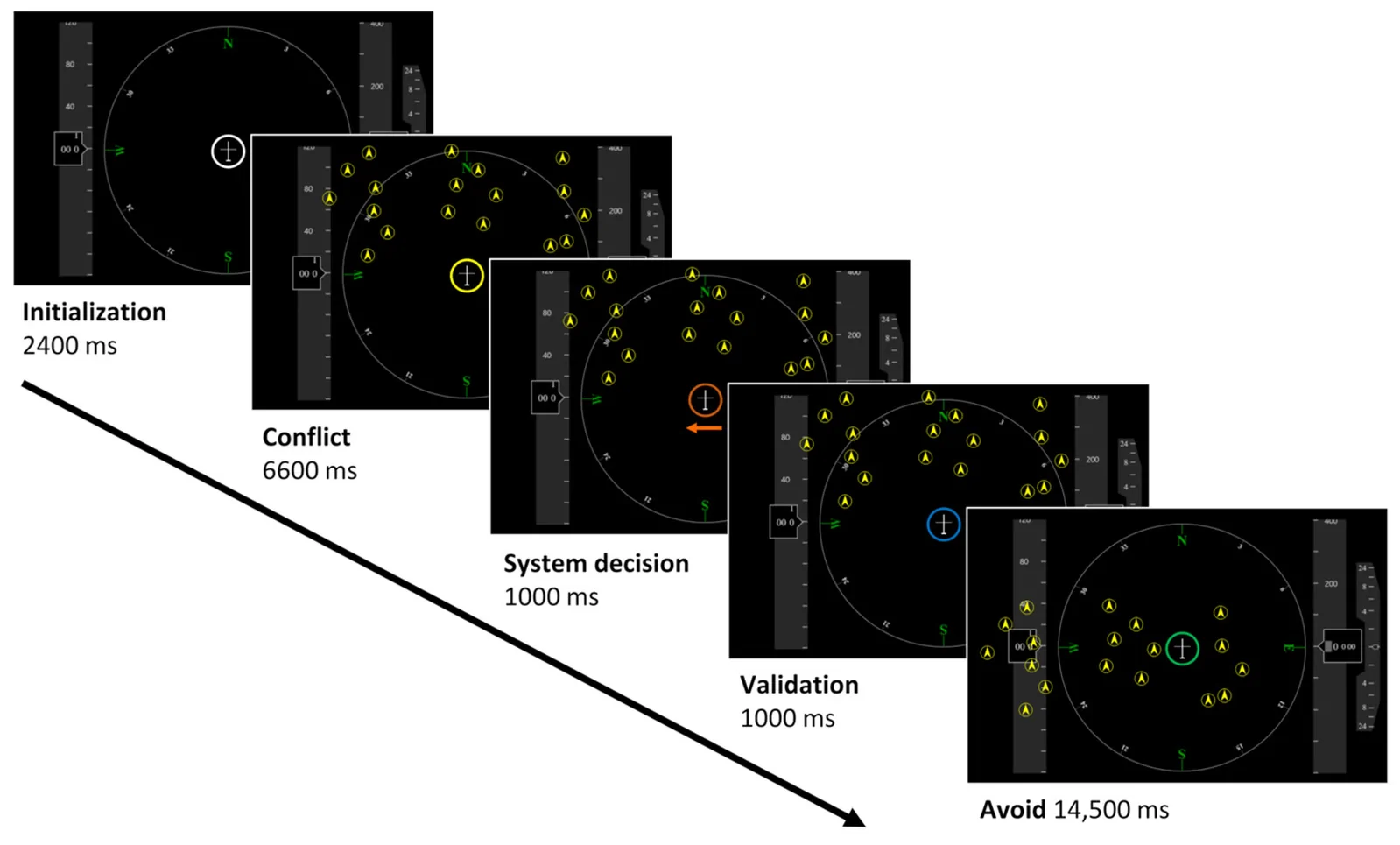
Chronological sequence of a trial.

**Figure 2:**
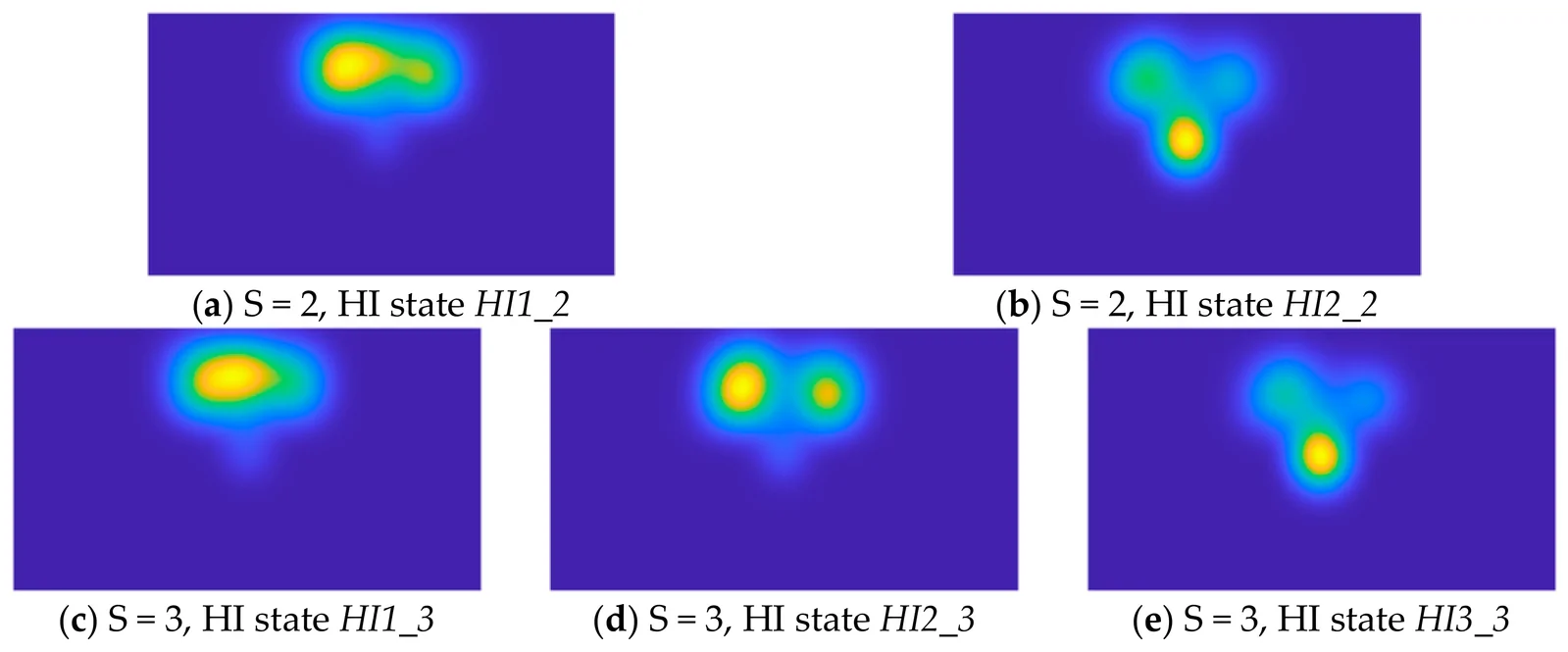
Experimental saliency map for each HI state. Color scale indicates fixation density, from low (blue) to high (yellow).

**Figure 3:**
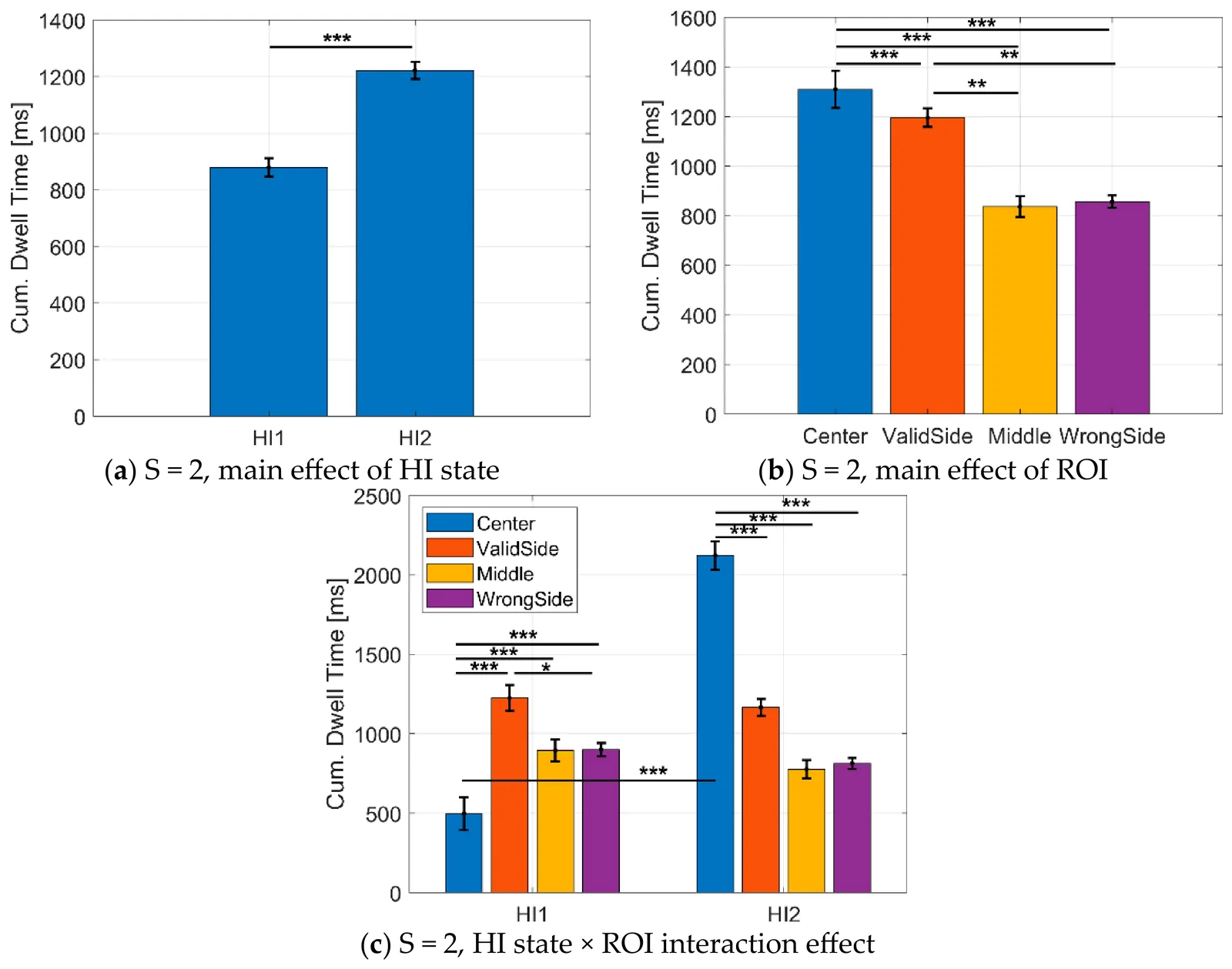
Mean cumulative dwell time (±SE) obtained from the two-HI-state SHMM showing (**a**) the main effect of HI state, (**b**) the main effect of ROI, and (**c**) the HI × ROI interaction. Bonferroni corrected *p*, * *p* < 0.05, ** *p* < 0.01, and *** *p* < 0.001.

**Figure 4:**
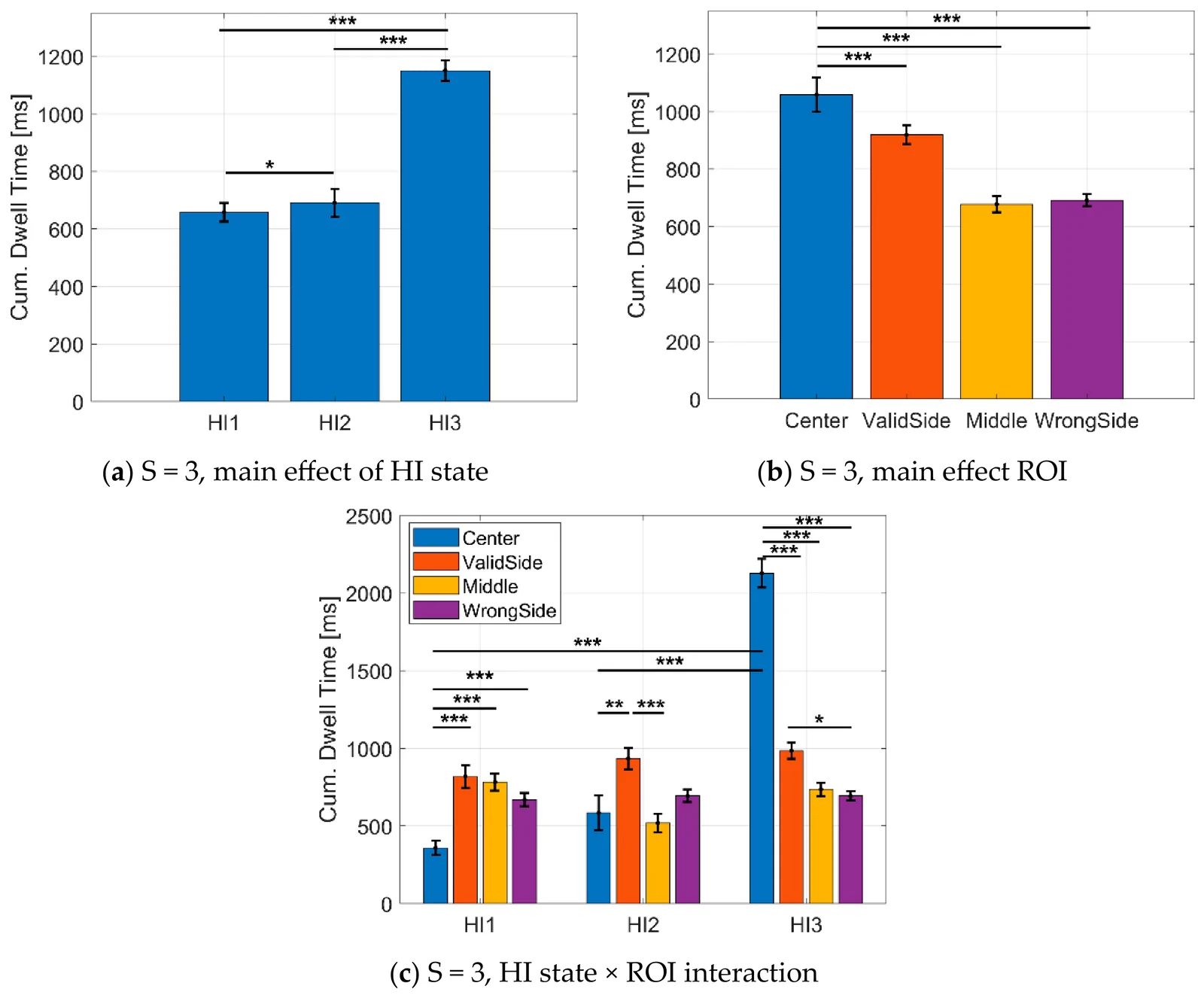
Mean cumulative dwell time (± SE) obtained from the three-HI-state SHMM showing (**a**) the main effect of HI state, (**b**) the main effect of ROI, and (**c**) the HI × ROI interaction. Bonferroni corrected *p*, * *p* < 0.05, ** *p* < 0.01, and *** *p* < 0.001.

**Figure 5:**
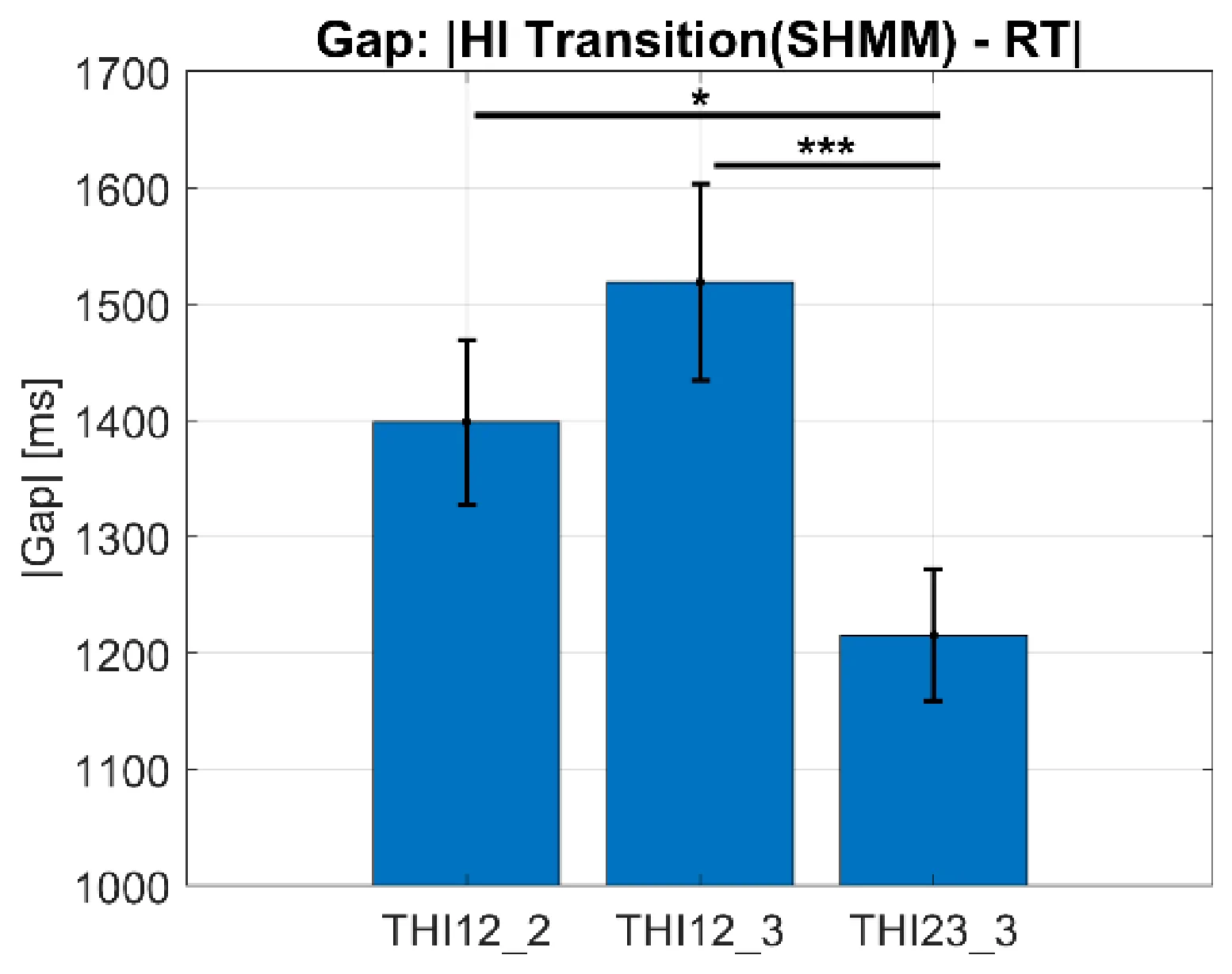
Mean (± SE) of the absolute gap, measured as the difference between RT and transition time, as a function of transition type, based on individual means. * *p* < 0.05, *** *p* < 0.001.

**Table 1:** Summary of initialization and configuration parameters for SHMMs.

Configuration\Initialization	S = 2, R = 4	S = 3, R = 4
Prior probabilities of HI states	[1 0]	[1 0 0]
HI state transition matrix	$\left(\begin{array}{cc} 0.8 & 0.2\\ 0 & 1 \end{array}\right)$	$\left(\begin{array}{ccc} 0.8 & 0.2 & 0\\ 0 & 0.8 & 0.2\\ 0 & 0 & 1 \end{array}\right)$
ROI transition matrix	Random	
Means and variances (ROIs), Gaussian density	From KMeans (K = R)	
Stopping criteria	Log-Likelihood decrease < 1.10^−5^; max 10,000 iterations	

**Table 2:** Post-hoc comparisons of KL divergences between the HI states (mean ± SE, F(1, 260) statistics, and corrected *p*-values). Significant differences are indicated in bold.

	Group 1: Final States		Group 2: Early States		
Mean ± SE	HI2_2	HI3_3	HI1_2	HI1_3	HI2_3
	0.14 ± 0.02	0.17 ± 0.02	0.32 ± 0.03	0.39 ± 0.03	0.55 ± 0.04
HI2_2	-	1.48, *p* = 1.00	**19.82, *p* < 0.001**	**29.37, *p* < 0.0001**	**37.58, *p* < 0.0001**
HI3_3	-	-	**10.47, *p* < 0.05**	**17.67, *p* < 0.001**	**24.16, *p* < 0.0001**
HI1_2	-			0.93, *p* = 1.00	2.82, *p* = 0.94
HI1_3	-	-	-	-	0.51, *p* = 1.00
HI2_3	-	-	-	-	-

**Table 3:** Descriptive statistics of the gap distributions between the adjacent HI state transition times and reaction times (RTs), for the three distinct transition time estimates.

Transition	Average [ms]	Std [ms]	Skewness	Kurtosis
THI12_2	−38.99	1711.88	−0.21	2.99
THI12_3	−1104.06	1442.99	0.04	3.25
THI23_3	392.30	1463.19	−0.16	3.30

**Table 4:** Fit and quality parameters (adjusted coefficient of determination, residual standard deviation, AIC, and log-likelihood) of the LMMs that explain reaction time (RT) by transition time (THI12_2, THI12_3, or THI23_3). For the best fit obtained with THI23_3 as the explanatory variable (in bold in the table), the average log-likelihood of state restoration by the SHMM for each trial (AvgLL_3) was added as an additional explanatory variable. The model including both a random intercept and a random slope for AvgLL_3 could not be reliably estimated (due to a near-singular covariance matrix) and is therefore not reported.

Transition	Parameters	Fixed Effect	Random Effect: Intercept	Random Effect: Intercept & Slope
THI12_2	β_0_ ± SE [ms]	2713.1 ± 38.491	2868.5 ± 133.44	2851.1 ± 144.55
	β_1_ ± SE	0.1221 ± 0.0115	0.0912 ± 0.0080	0.0947 ± 0.0152
	Marginal R^2^	0.0212	0.0119	0.0129
	Conditional R^2^	0.0212	0.6045	0.6154
	Residual std	1176.1	748.39	738.66
	AIC	89,122	84,646	84,575
	Log likelihood	−44,558	−42,319	−42,282
THI12_3	β_0_ ± SE [ms]	2692 ± 34.412	2926.6 ± 131.89	2920.5 ± 169.51
	β_1_ ± SE	0.1984 ± 0.0153	0.1106 ± 0.0110	0.1178 ± 0.0194
	Marginal R^2^	0.0309	0.0097	0.0110
	Conditional R^2^	0.0309	0.6023	0.6103
	Residual std	1170.2	750.43	743.14
	AIC	89,070	84,673	84,648
	Log likelihood	−44,532	−42,333	−42,318
THI23_3	β_0_ ± SE [ms]	2182.3 ± 47.388	2567.9 ± 131.75	2531 ± 144.58
	β_1_ ± SE	0.2596 ± 0.0128	0.1659 ± 0.0096	0.1699 ± 0.0183
	Marginal R^2^	0.0722	0.0297	0.0313
	Conditional R^2^	0.0722	0.6166	0.6282
	Residual std	1145	736.89	726.35
	AIC	88,841	84,482	84,408
	Log likelihood	−44,418	−42,237	−42,198
THI23_3 + AvgLL_3	β_0_ ± SE [ms]	516.19± 249.53	**1507.10 ± 217.83**	
	β_1_ ± SE	0.2517± 0.0128	**0.1570 ± 0.0097**	
	β_2_ ± SE	−269.85 ± 39.69	**−173.76 ± 28.48**	
	Marginal R^2^	0.0803	**0.0317**	
	Conditional R^2^	0.0803	**0.6193**	Instable model
	Residual std	1140.0	**734.29**	
	AIC	88,797	**84** **,** **447**	
	Log likelihood	−44,395	**−42** **,** **219**	

## Data Availability

The data supporting the findings of this study are available at https://gricad-gitlab.univ-grenoble-alpes.fr/salomomi/JEMR_AGD_SHMM/.

## Appendix A

As detailed in Section 3.1.1, the two- and three-state HI configurations were chosen a priori based on theoretical considerations rather than through data-driven model selection. For completeness, however, we report here a quantitative comparison of the two retained configurations (S = 2, and S = 3), computed using the Akaike Information Criterion (AIC) for each participant’s individual SHMM. AIC values were compared using a random-intercept LMM. The analysis revealed a statistically significant advantage for the three-state model (F(1, 104) = 55.14, *p* < 0.0001; mean AIC ± SE = 21,542 ± 561 for S = 3 vs. 21,581 ± 562 for S = 2), which confirms the statistical justification of the three-state model’s increased complexity. However, we believe this should not be interpreted as validating one configuration over the other, or supporting the forward-only transition structure itself, since no less constrained alternative transition structures were examined. As discussed in Section 4.4 and Section 4.5, the two configurations capture complementary aspects of the same decision-making process and are not competing to be a single “best” description.
